# Should we offer preventive treatment to all carriers of *PLN* p.(Arg14del) variant? *Pro:* Provide pre-emptive treatment to asymptomatic carriers

**DOI:** 10.1007/s12471-023-01793-0

**Published:** 2023-07-26

**Authors:** Rudolf A. de Boer

**Affiliations:** Department of Cardiology, Erasmus University Medical Centre, Rotterdam, The Netherlands

Although I am an optimist by nature, phospholamban (*PLN*) p.(Arg14del) cardiomyopathy is a highly malignant condition that must be avoided at all costs. In carriers of the *PLN* p.(Arg14del) variant in their 30s to 50s, the risk of sudden cardiac death (SCD) is approximately 10-fold higher than that in age-matched non-carriers [1, 2]. Moreover, a sizeable proportion of the Dutch patients receiving a heart transplantation suffer from *PLN* p.(Arg14del) cardiomyopathy. Not only do *PLN* p.(Arg14del) carriers face these factual risks, if they develop the disease, they also enter a cycle of progressive disease [1, 2]. Many carriers—who are usually in the prime of their lives—end up being a diseased citizen, with intrinsic uncertainties, declining quality of life, potential loss of job and hobbies and reliance on social and medical networks, resulting in many years lost. We know *PLN* p.(Arg14del) cardiomyopathy is an autonomically progressive disorder with protein aggregates, left ventricular (LV) fibrosis and dysfunction [3, 4], features that cannot be reversed. Waiting for LV scarring and ventricular arrhythmia will therefore result in longstanding severe symptoms and irreparable damage. I believe we as doctors must at least try to keep these patients free of disease!

The major question is: does pre-emptive medication slow down the onset of this disease? We recently concluded the Dutch iPHORECAST study, which compared the mineralocorticoid receptor antagonist eplerenone with control treatment (e.g. no medication) in asymptomatic * PLN* p.(Arg14del) carriers [5]. The study showed that the rate of progression is alarming, but it not so easy to slow this down. In an experimental mouse model of *PLN* cardiomyopathy, the mice did not respond to established heart failure (HF) drugs [3]. However, since an experimental tailored therapy was very effective [6], I am confident we can target the abnormal *PLN* within a reasonable time span. For the time being, I trust common HF drugs will provide some protection.

So, what is the hang-up with pre-emptive treatment exactly? In cardiomyopathy care, we generally accept a 5% risk for SCD as sufficient to implant a prophylactic intracardiac defibrillator. In HF care, we generally accept a relative risk reduction of 20% for any drug treatment to be eligible, which, depending on the absolute risk, generally translates into an absolute risk reduction of 2–5% to reduce one cardiovascular death or HF hospitalisation in 2–3 years, with a number needed to treat of 20–30. Even in primary cardiovascular prevention, we consider a 10-year risk of major adverse cardiovascular events of 10–20% to be sufficient to start primary preventive pharmacotherapy. For *PLN* p.(Arg14del) cardiomyopathy, the numbers are stunning: there is a seemingly 50% lifetime risk to develop severe disease, i.e. SCD, ventricular tachycardia or HF. This risk is far higher than that in other conditions for which we do initiate pre-emptive treatment. Side effects of angiotensin-converting enzyme inhibition and angiotensin receptor blockers are minimal, or it must be they have been associated with less stroke, incident HF and incident renal disease. One wonders what the cutpoint (percentage) to initiate pre-emptive treatment is for Pieter Doevendans if 50% is still not enough. Should it be 100% instead?

If an asymptomatic carrier shows up in my outpatient clinic at the age of 45 with a typical microvoltage electrocardiogram [7] but a still normal magnetic resonance imaging scan, with minimal ventricular ectopy, I will congratulate him/her for still being on the good side of things. But I will not hesitate to offer pre-emptive medication.
